# Supplementary material for: Floquet non-Abelian topological insulator and multifold bulk-edge correspondence
Source: Nat Commun. 2023 Oct 12;14:6418. doi: 10.1038/s41467-023-42139-z (PMC10570273; doi:10.1038/s41467-023-42139-z)
Supplement: Supplementary file 1 — Supplementary Information [file 41467_2023_42139_MOESM1_ESM.pdf]

Supplemental Material for "Floquet Non-Abelian Topological Insulator and Multifold Bulk-Edge Correspondence"

This supplemental material provides additional details on the multiplication rule of Dirac singularities in the phase band, examples with multiple Dirac touchings in the same gap, the phase-band picture of the interface effect, the quotient relation in domain-wall problem, the Zak phase of quasienergy bands, and the stability of edge/domain-wall states.

Appendix A: Multiplication rule of Dirac singularities

In the main text, we have established a relationship between the quaternion charge q of the Floquet Hamiltonian and the charges of the Dirac singularities. This is expressed through the equation:

$$q = \prod_m \tilde{q}_m, \tag{A1}$$

In the equation, we stipulate that the multiplication is from left to right. Since the quaternion group Q_8 is non-Abelian, the ordering in the product is important. We use Fig. S1 to illustrate our choice of ordering in the 2D momentum-time space. Firstly, all the enclosing paths of the Dirac points share the same starting point P. Secondly, the composition of these paths, i.e., the concatenation of paths in the fundamental group, should be smoothly deformed to the boundaries of the 2D (k,t) space clockwise. For example, in Fig. S1, the path concatenation associated with the multiplication $\tilde{q}_1 \cdot \tilde{q}_2 \cdot ... \cdot \tilde{q}_n$ is equivalent to circling around the boundaries of the 2D (k,t) space clockwise. On the other hand, the path concatenation associated with the multiplication $\tilde{q}_n \cdot \tilde{q}_{n-1} \cdot ... \cdot \tilde{q}_2 \cdot \tilde{q}_1$ is equivalent to circling around the boundaries counterclockwise. Notably, the former coincides with the integral taken from $-\pi$ to π in calculating the quaternion charge of the Floquet Hamiltonian.

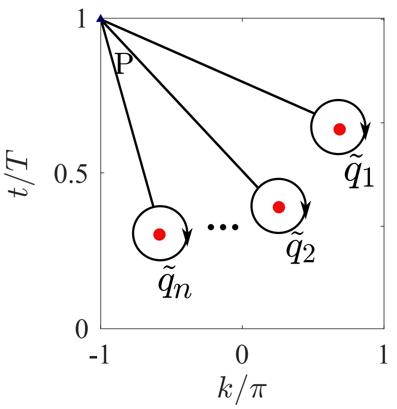

FIG. S1. Path concatenation and ordering of Dirac singularities. The quarternion charge q of the Floquet Hamiltonian is related to the charges of the singularities through $q = \tilde{q}_1 \cdot \tilde{q}_2 \cdot ... \cdot \tilde{q}_n$.

Appendix B: Examples with multiple phase-band touchings in the same gap

In the main text, we have listed all the phase-band patterns with at most one touching in each gap in Fig. 3. In principle, multiple touchings within the same gap are possible, as demonstrated by two such cases in Fig. S2**a** and **b**. Specifically, in Fig. S2**a**, the quaternion charge of the bulk Floquet Hamiltonian and the Dirac points are related through $i = -\tilde{k} \cdot \tilde{k} \cdot \tilde{i}$, while in Fig. S2**b**, they are related through $-1 = (-\tilde{i}) \cdot \tilde{k} \cdot (-\tilde{i}) \cdot \tilde{k}$. Furthermore, we have plotted the corresponding quasienergy spectra and spatial distributions of eigenstates under open boundary conditions in Figs. S2**c** and **d**. For the former, we observe the existence of a single edge state in the second gap. For the latter, we observe a pair of edge states for the first and second gap, respectively.

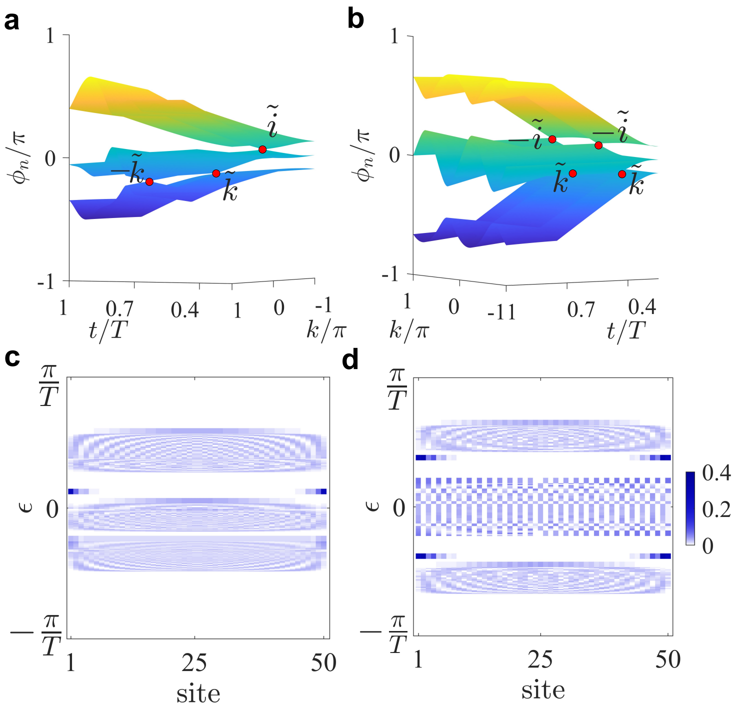

FIG. S2. Phase bands with multiple touchings in the same gap. **a** Phase bands with bulk quaternion charge $q = i$ for the Floquet Hamiltonian. It is related to the charges of the Dirac points through $i = -\tilde{k}\cdot\tilde{k}\cdot\tilde{i}$. **b** Phase bands with bulk quaternion charge $q = -1$ for the Floquet Hamiltonian. It is related to the charges of the Dirac points through $-1 = (-\tilde{i})\cdot\tilde{k}\cdot(-\tilde{i})\cdot\tilde{k}$. **c**, **d** Quasienergy spectra and the spatial distributions of eigenstates with open boundaries corresponding to the phase bands in **a**, **b**, respectively. The lattice length is $L = 50$. The parameters are listed in Methods.

Appendix C: Interface effect from the phase-band picture

An alternative explanation of the emergence of interface modes is from the phase-band picture. The bulk Floquet operators on the left and right sides are $U_L = e^{-iH_1T/4}e^{-iH_2T/2}e^{-iH_1T/4}$ and $U_R = e^{-iH_2T/4}e^{-iH_1T/2}e^{-iH_2T/4}$, respectively. We denote their bulk quaternion charges as q_L and q_R. Let us formally consider the two PT symmetric operators as below:

$$\tilde{U}^{(1)}(k,t) = \begin{cases} e^{-iH_1t}, & t\in[0,1/2],\\ e^{-iH_1/4}e^{-iH_2(t-1/2)}e^{-iH_1/4}, & t\in[1/2,1], \end{cases} \tag{C1}$$

$$\tilde{U}^{(2)}(k,t) = \begin{cases} e^{-iH_1t}, & t\in[0,1/2],\\ e^{-iH_2(t-1/2)/2}e^{-iH_1/2}e^{-iH_2(t-1/2)/2}, & t\in[1/2,1]. \end{cases} \tag{C2}$$

$\tilde{U}^{(1)}(k,t)$ and $\tilde{U}^{(2)}(k,t)$ are related by an $SO(3)$ transform, and thus have the same phase bands. At $t = T$, the phase bands of $\tilde{U}^{(1)}(k,T)$ and $\tilde{U}^{(2)}(k,T)$ coincide with the bulk quasienergy bands of U_L and U_R, respectively:

$$\tilde{U}^{(1)}(k,T) = U_L, \quad \tilde{U}^{(2)}(k,T) = U_R. \tag{C3}$$

Our numerical analysis shows that there are three Dirac singularities with charges $\tilde{k}$, $-\tilde{i}$, and $\tilde{j}$ for $\tilde{U}^{(1)}(k,t)$ and three Dirac singularities with charges $-\tilde{k}$, $\tilde{i}$, and $-\tilde{j}$ for $\tilde{U}^{(2)}(k,t)$. According to Eq. (A1), the quaternion charge of the bulk Floquet Hamiltonian is $q_L = \tilde{k}\cdot(-\tilde{i})\cdot\tilde{j} = 1$, and $q_R = -\tilde{k}\cdot\tilde{i}\cdot(-\tilde{j}) = -1$. Therefore, domain-wall states are expected to emerge at the interface.

Appendix D: Quotient relation in the domain-wall problem

In this section, we address the domain-wall problem for Floquet non-Abelian topological insulators (FNATIs). As depicted in Fig. S3**a**, let us denote q_L and q_R as the bulk quaternion charge of the Floquet Hamiltonian on the left

and right side, respectively. In the static case, the appearance of domain-wall states between two topologically distinct phases is governed by the quotient relation $\Delta q = q_L/q_R$. We demonstrate that in the Floquet setting, the quotient relation still holds. However, due to the additional bandgap across the Floquet Brillouin zone edge, the quotient relation implies a multifold bulk-domain-wall correspondence. For specific values of q_L and q_R, their quotient Δq is definite, but the domain-wall states may exhibit different configurations, as summarized in Fig. 3 of the main text. In fact, the edge-state patterns listed in Fig. 3 can be seen as a special case of the domain-wall problem, where the system interfaces with the vacuum. Considering its multifold nature, determining the domain-wall states requires additional information about the phase-band singularities. These singularities are related to the bulk quaternion charge through $q_L = \tilde{q}_{L1}\tilde{q}_{L2}...$ and $q_R = \tilde{q}_{R1}\tilde{q}_{R2}...$, respectively. We proceed to demonstrate the quotient relation using three steps.

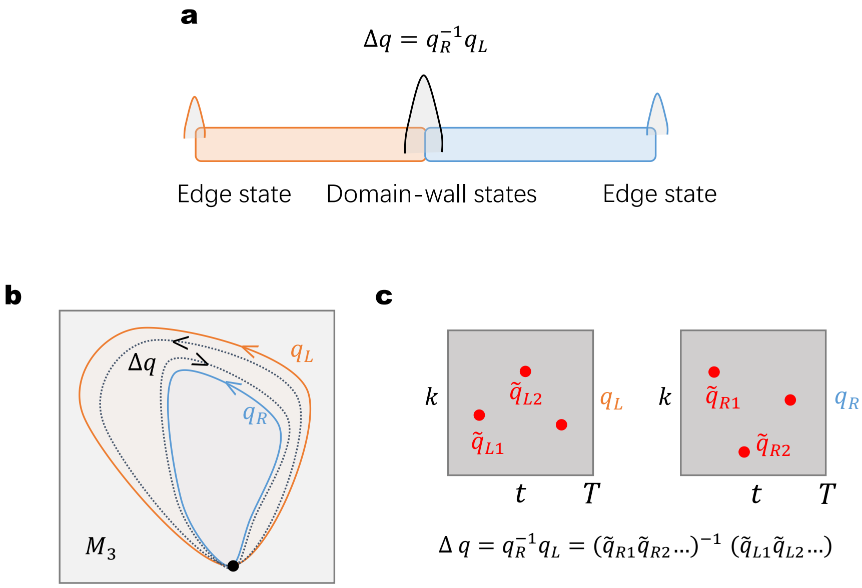

FIG. S3. Quotient relation of Floquet non-Abelian topological insulators (FNATIs). **a** Sketch of the domain wall, where the left and right sides correspond to bulk Hamiltonians with quaternion charges q_L and q_R, respectively. The domain wall is described by the quotient relation $\Delta q = q_R^{-1} q_L$. **b** Geometric visualization of the quaternion charge as a closed path (loop) in the configuration space M_3. The black dot is the base point. The change in quaternion charge from q_R (blue) to q_L (orange) is given by Δq (dotted black loop), and the concatenated path $q_R \Delta q$ is equivalent to q_L. **c** Relationship between the quaternion charge and the phase-band singularities.

Firstly, between two distinct topological phases, there are bandgap closings at the domain wall, corresponding to the topological phase transition. This holds true for both static and Floquet cases. Importantly, in Floquet systems, two systems being topologically distinct implies different phase-band singularities or edge-state patterns. In Fig. 3 of the main text, for the same quaternion charge, there may be multiple distinct phases due to their different phase-band singularities.

Secondly, the details of the bandgap closings, such as their position and times, are encoded in the quotient relation $\Delta q = q_R^{-1} q_L$. (Here q_L/q_R is treated as $q_R^{-1} q_L$; the other choice $q_L q_R^{-1}$ is also fine as they are conjugate). To visualize this, we remind that the quaternion charge is the first homotopy invariant of the configuration space $M_3 = \frac{O(3)}{O(1)^3}$ of PT-symmetric Hamiltonians. Geometrically, the quaternion charge can be represented as a closed path in M_3 space, as depicted in Fig. S3**b**. At the domain wall, the quaternion charge undergoes a change from q_R to q_L through gap closings. This change is described by an intermediate path (the dotted black loop) denoted as Δq. In homotopy language, concatenating the two paths $q_R \Delta q$, first following q_R and then Δq, yields the path q_L: $q_L = q_R \Delta q$. Thus, we have $\Delta q = q_R^{-1} q_L$. In Floquet systems, the quaternion charge on each side is further related to the phase-band singularities through Eq. (4) in the main text, as sketched in Fig. S3**c**. Consequently, we have $\Delta q = q_R^{-1} q_L = (\tilde{q}_{R1}\tilde{q}_{R2}...)^{-1}(\tilde{q}_{L1}\tilde{q}_{L2}...)$.

Thirdly, the gap closings recorded in the Δq above give rise to domain-wall states through the Jackiw-Rebbi argument [1]: the number and locations of the gap closings determine the number of edge states in that gap. In other words, whenever there is a gap closing, a domain-wall state appears. By accounting for all gap closings inside Δq represented by the phase-band singularities, we can determine the patterns of domain-wall states.

To compare with the static case and demonstrate the multifold nature of Floquet systems, let us consider a domain wall between two Floquet Hamiltonians of the same charge, $q_L = q_R = j$, as shown in Fig. S4. The quotient is $\Delta q = 1$.

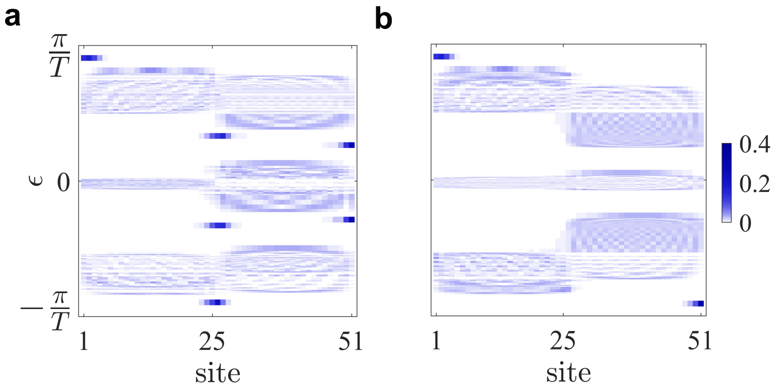

FIG. S4. Quasienergy spectra and spatial distributions of the eigenstates in the presence of a domain wall. The bulk quaternion charge for both sides is $q_L = q_R = j$. **a** A domain wall between $q_L = j$ (with singularity $\tilde{j}$) and $q_R = j$ (with singularity $\tilde{k}\tilde{i}$) possesses three domain-wall states (one in each gap), as per $\Delta q = 1 = (\tilde{i}\tilde{k})\tilde{j}$. **b** A domain wall between $q_L = j$ (with singularity $\tilde{j}$) and $q_R = j$ (with singularity $\tilde{j}$) does not possess any domain-wall state.

The domain wall between $q_L = j$ (with singularity $\tilde{j}$) and $q_R = j$ (with singularity $\tilde{k}\tilde{i}$) possesses three domain-wall states as $\Delta q = (\tilde{i}\tilde{k})\tilde{j}$ [See Fig. S4**a**]. On the other hand, the domain wall between $q_L = j$ (with singularity $\tilde{j}$) and $q_R = j$ (with singularity $\tilde{j}$) possesses no domain-wall state [See Fig. S4**b**]. The two different configurations are consistent with the charge 1 class listed in Fig. 3 of the main text.

Appendix E: Zak phase of the quasienergy band

Besides the quaternion charge, the Zak phase is another topological invariant that is assigned to each quasienergy band. The Zak phase can take two values, 0 or π. To calculate the Zak phase, we diagonalize the Floquet Hamiltonian $H_{\mathrm{F}}(k) = S(k)\mathrm{diag}(\epsilon_1, \epsilon_2, \epsilon_3)S^T(k)$. Here, the eigenvectors are organized in the $SO(3)$ transformation $S(k) = (|u_1\rangle, |u_2\rangle, |u_3\rangle)$. The Zak phase can be extracted from the evolution of these eigenvectors with respect to the lattice momentum k. As k ranges from $-\pi$ to π, the eigenvector $|u_j\rangle$ ($j = 1, 2, 3$) may acquire an additional sign in a continuous manner, $|u_j(k = \pi)\rangle = -|u_j(k = -\pi)\rangle$. This corresponds to the Zak phase taking π for the j-th band. Otherwise, the Zak phase takes 0. Formally, one can consider the matrix $S(\pi)^T S(-\pi)$, which takes the form of $\mathrm{diag}(\lambda_1, \lambda_2, \lambda_3)$. λ_j ($j = 1, 2, 3$) $= \pm 1$. The Zak phase of the j-th band is 0 (π) if $\lambda_j = 1$ ($\lambda_j = -1$) [2]. It should be noted that in the static case [3], the Zak phase also takes other values, such as $-\pi$ and 2π. They are introduced solely to distinguish between two conjugate elements in the same class. $\pm\pi$ are associated with different gauge choices of the eigenstates, and treated as the same here. We list the obtained Zak phases for all quaternion charges below:

TABLE S1. Zak phase of the quasienergy band

q	1	$\{\pm i\}$	$\{\pm j\}$	$\{\pm k\}$	-1
Third band	0	π	π	0	0
Second band	0	π	0	π	0
First band	0	0	π	π	0

In the same conjugacy class (e.g., i and $-i$), the Zak phase pattern is the same. The constraint that the summation of Zak phases for all three bands equals 0 (mod 2π) leads to four possible Zak phase patterns. Since there are five conjugacy classes in the quaternion group Q, the class -1 falls outside the scope of the Zak-phase description. Furthermore, the Zak phase is defined for the Floquet Hamiltonian, while the topology of the system is fully encoded in the time-evolution operator or phase-band singularities. As discussed in the main text, the bulk-edge (or domain-wall) correspondence is multifold. Different patterns of edge states (domain-wall states) can have the same quaternion charge and Zak phase configuration. Therefore, the quaternion charge or the Zak phase is insufficient to predict the edge states, and descriptions based on phase-band singularities are necessary.

Appendix F: Stability of edge/domain-wall states

In this section, we conduct numerical studies to test the stability of edge states and domain-wall states. We start by varying the boundary conditions and showcase the emergence of edge states. Starting from the periodic boundary condition, we gradually decrease the coupling strengths between the first and the last (N-th) unit cell: $v_{XY,N} = (1-\alpha)v_{XY}$. Here v_{XY} represents the intercell coupling in the bulk [See the model, Eq. (1) in the main text], and $v_{XY,N}$ denotes the intercell coupling between the first and the last unit cell. In Fig. S5**a**, **b**, we plot the quasienergy bands for quaternion charges i (with phase-band singularity $\tilde{i}$) and k (with phase-band singularity $\tilde{k}$), respectively. By varying α continuously from 0 (periodic boundary condition) to 1 (open boundary condition), we observe the appearance of edge states in the second gap for charge i and the first gap for charge k, detached from neighboring bands.

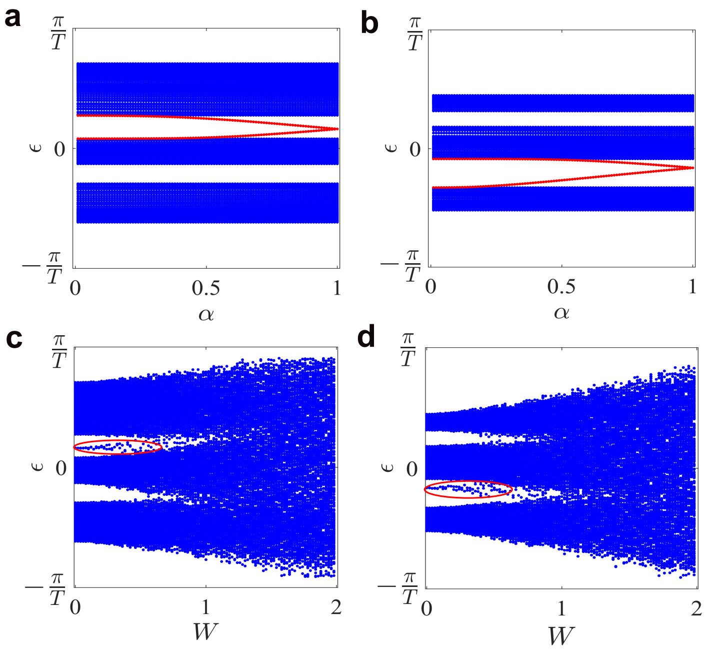

FIG. S5. Stability of edge states in the presence of disorder. **a, b** Quasienergy spectra for quaternion charges i and k as a function of the boundary coupling strength α. The intercell coupling across the boundary is $v_{XY,N} = (1-\alpha)v_{XY}$, where $\alpha = 0$ and $\alpha = 1$ correspond to periodic and open boundary conditions, respectively. The in-gap edge modes (red lines) emerge from the neighboring bulk bands. **c, d** Quasienergy spectra under open boundary condition as a function of the disorder strength W. The edge states are encircled by red ellipses. The parameters used are listed in Table 1.

We proceed to examine the robustness of the edge states by introducing onsite disorder: $s_{XX,n} = s_{XX} + \delta$ ($n = 1, 2, \ldots N, X = A, B, C$). Here, δ is taken from the uniform distribution of $[-W, W]$, with W representing the disorder strength. The numerical results are presented in Fig. S5**c**, **d**. It is evident that the edge states persist as long as the bulk quasienergy gaps are not closed by the disorder.

To test the stability of the domain-wall states, we consider four different types of domain walls, as depicted in Fig. S6**a-d**. In Fig. S6**a**, **b**, the intracell couplings s_{XY} of the interface unit cell are set to be the same as those on the left and right sides, respectively. In Fig. S6**c**, **d**, the intracell couplings are set to zero for the interface unit cell, except for the additional next-nearest-neighbor couplings (with strength $v = 0.5$) in the latter case. We set the left/right side to possess quaternion charges i (with phase-band singularity $\tilde{i}$) and k (with phase-band singularity $\tilde{k}$) as an example. For all four types of domain wall, we observe the emergence of domain-wall states in the first and second gaps, as shown in Fig. S6**e-h**. This is consistent with the quotient relation, $\Delta q = q_R^{-1} q_L = -\tilde{k}\tilde{i}$. Furthermore, we test the robustness of these domain-wall states against onsite disorder in Fig. S6**i-l**. Similar to the edge states at the boundary, the domain-wall states survive weak disorder.

It is worth mentioning that the decorations of the domain wall may induce some additional bound states, as illustrated in Fig. S7. We consider a tunable onsite potential with strength p at the interface unit cell: $s_{AA} = s_{BB} = s_{CC} = p$. In Fig. S7**a** to **d**, we take four values of $p = 3, 9, 50, 1000$ and plot the quasienergy spectra and spatial distributions of the eigenstates. The domain-wall states located in the first and second gaps always exist. However, there also appear some trivial bound states. These bound states have no topological origin. Their quasienergies depend

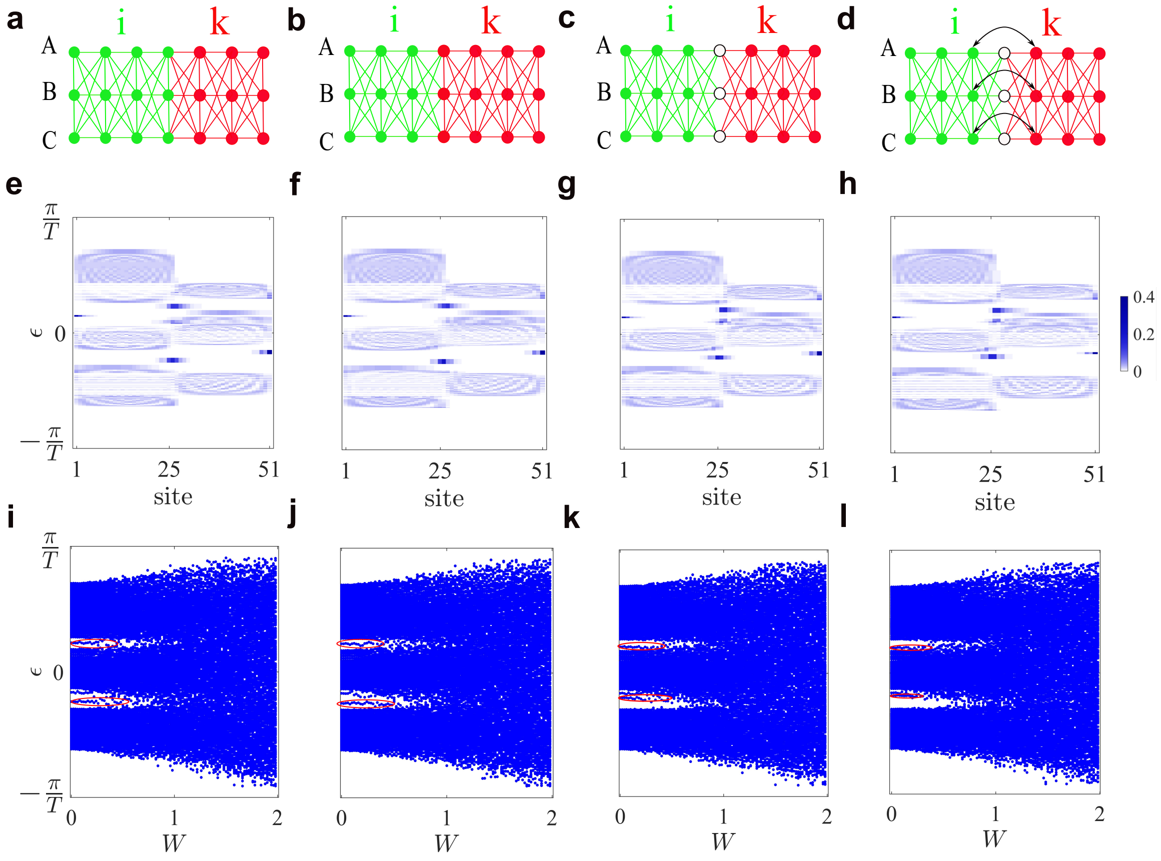

FIG. S6. Stability of domain-wall states against different domain-wall settings and disorder. **a-d** Domain-wall configurations. The left (right) side has bulk quaternion charge i (k). The black arrows in **d** indicate the next-nearest-neighbor couplings. **e-h** Quasienergy spectra and spatial distributions of eigenstates for the four domain-wall settings. **i-l** Quasienergy spectra as a function of the disorder strength W. The domain-wall states are encircled by red ellipses. The parameters used are listed in Table 1.

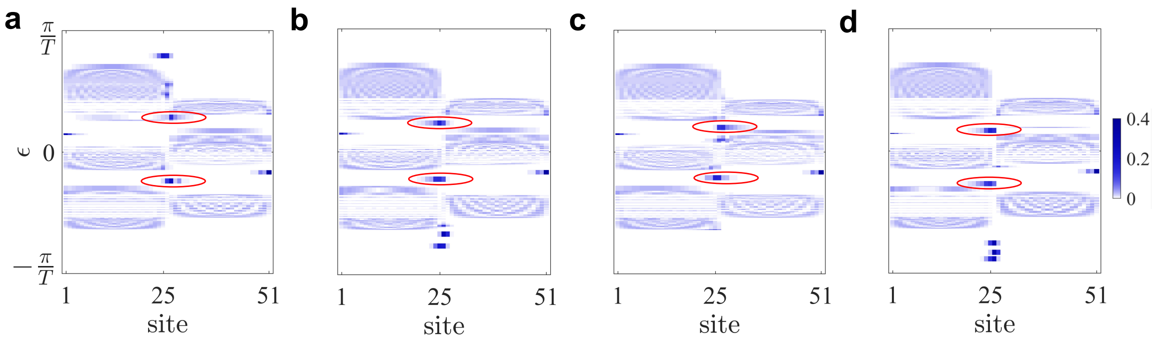

FIG. S7. Quasienergy spectra and spatial distributions of eigenstates for different onsite potentials at the interface unit cell. (a-d) The potential strengths are $p = 3, 9, 50$ and 1000, respectively. The domain-wall states in the first and second gaps always exist (encircled by red ellipses). Trivial bound states may arise due to the local onsite potential. The parameters used are listed in Table 1.

on the added potential and may merge into the bulk bands by adjusting the potential. In contrast, the domain-wall

states are robust. They originate from the non-Abelian topology and are described by the quotient relation.

[1] S. -Q. Shen, *Topological insulators* , pages 19-22 (Berlin: Springer, 2012).
[2] T. Jiang, Q. Guo, R.-Y. Zhang, Z.-Q. Zhang, B. Yang and C. T. Chan, Four-band non-Abelian topological insulator and its experimental realization. Nat. Commun. **12**, 6471 (2021). doi: 10.1038/s41467-021-26763-1
[3] Q. Guo, T. Jiang, R.-Y. Zhang, L. Zhang, Z.-Q. Zhang, B. Yang, S. Zhang and C. T. Chan, Experimental observation of non-Abelian topological charges and edge states. Nature **594**, 7862 (2021). doi: 10.1038/s41586-021-03521-3
